# Prognostic communication in heart failure: protocol for a systematic qualitative synthesis of experiences, attitudes and practices

**DOI:** 10.1136/bmjopen-2025-099088

**Published:** 2025-11-04

**Authors:** Christina Chu, Steven Bloch, Sarah Yardley

**Affiliations:** 1 Marie Curie Palliative Care Research Department, University College London, London, UK; 2 Language and Cognition, University College London, London, UK

**Keywords:** Prognosis, Heart failure, Person-Centered Care, Adult palliative care

## Abstract

**Abstract:**

**Introduction:**

Being able to talk about the anticipated course of living with an illness is essential to delivering and receiving person-centred care. Despite clinical heart failure guidance encouraging these prognostic conversations at all stages of disease, they occur infrequently or very late in the disease course. This qualitative synthesis will use the Joanna Briggs Institute (JBI) meta-aggregation approach to explore how prognostic conversations are currently taking place, what people think about prognostic conversations, and how people experience them.

**Methods and analysis:**

This systematically conducted qualitative synthesis, using JBI meta-aggregation, considers qualitative evidence that explores the prognostic communication experiences, attitudes or practices of people with heart failure and their healthcare professionals. Prognostic communication is defined as a verbal interaction about anticipated changes to symptoms or function, possibility of unpredictable events, potential future treatments or care, expression of wishes about the future, or estimates of life expectancy. It will include interactions occurring in any setting (inpatient, outpatient, community). Exclusion criteria include studies of carer perspectives, discussion about implantable cardiac defibrillator deactivation, assisted dying and/or euthanasia, and those not published in the English language. Medical Literature Analysis and Retrieval System Online (MEDLINE) (Ovid), Excerpta Medica Database (EMBASE) (Ovid), PsycInfo (Ovid), Cumulative Index to Nursing and Allied Health Literature (CINAHL) Plus (EBSCOhost), Web of Science Core, Overton, ProQuest Dissertations and Theses Global, and Google Scholar databases will be searched for eligible studies. Reference screening of relevant systematic reviews will also be conducted. Two independent reviewers will screen, quality assess included studies and perform data extraction. JBI tools will be used for quality appraisal, data extraction, synthesis and assessing confidence of summarised findings.

**Ethics and dissemination:**

Ethical approval is not required for the study since it is based on available published literature. Findings from the review will be disseminated through publication in a peer-reviewed journal.

**Trial registration number:**

International Prospective Register of Systematic Reviews (PROSPERO) CRD42024605240.

**STRENGTHS AND LIMITATIONS OF THIS STUDY**

- This is the first systematic review to consider prognostic communication broadly, and not just limited to end of life care, in heart failure.
- Methodological rigour is achieved through use of Joanna Briggs Institute (JBI) meta-aggregation methodology for the systematic synthesis of qualitative evidence.
- The search strategy is comprehensive and includes articles identified from database and grey literature searches.
- The synthesis relies on the presented data and author interpretations of included studies, which may not reflect the full scope of the original data.
- This study is limited by exclusion of papers not published in the English language.

## Introduction

 Healthcare strategy has increasingly advocated for personalised care since the early 2000s.^1^ Advance care planning is one example, which involves personalised prognostic conversations that help patients understand how their condition may affect them, including changes in symptoms, ability to self-care and life expectancy.^2^ Effective prognostic conversations are therefore essential to person-centred care; they are complex and require sensitivity and responsiveness to the individual, a balance between optimism and realism, and maintainance of hope while acknowledging uncertainty.

Unfortunately, people with heart failure rarely have these conversations, or they occur late in their illness. Even if they do occur, patients often find their questions remain unanswered or are dissatisfied with how information was delivered.^3^ This contrasts sharply with heart failure guidelines, which encourage prognostic conversations throughout the disease course.^4^,^7^

Despite recognising the value of these conversations, their avoidance stems from concerns of both clinicians and patients.^3 8 9^ Clinicians are worried about time constraints, communication skills, the patient’s ability to understand and the impact on the patient’s mood and sense of hope. Patients fear making the clinician uncomfortable, or that their questioning may affect their care.

With heart failure 5-year and 10-year survival rates comparable to or worse than all cancers combined, and its growing prevalence, this is a significant problem.^10^,^13^ Without timely conversations, many people will miss opportunities to plan for their future.

To address this problem and change practice, we aim to examine current practices, perceptions and experiences. Examining the problem from this perspective could provide helpful insights.

To give us a rich understanding, we will systematically conduct a qualitative synthesis to answer the following question: what are the prognostic communication experiences, attitudes and practices of people with heart failure and their healthcare professionals?

A preliminary search of the International Prospective Register of Systematic Reviews (PROSPERO), Medical Literature Analysis and Retrieval System Online (MEDLINE), Cochrane Database of Systematic Reviews, and Joanna Briggs Institute (JBI) Evidence Synthesis found no existing or ongoing systematic reviews on this topic. Three related reviews were identified. Barclay *et al* explored ‘end of life conversations’ in heart failure.^8^ This review found diverse patient attitudes towards these conversations, and that end of life care was rarely discussed and difficult to introduce and navigate. A scoping review published in 2020 echoed these findings.^9^ A PROSPERO-registered review on advance care planning for patients with stage C or D heart failure, assessed by New York Heart Association (NYHA) classification II to IV, has no documented update since February 2023.^14^

In contrast to these previous reviews, our review will take a broader perspective on prognostic talk across all heart failure stages, aligning with guidelines that promote prognostic conversations at all stages throughout the disease. We will exclude discussions about deactivating implantable cardiac defibrillators (ICDs) in light of three recent reviews published on this topic.^15^,^17^ Similarly, these reviews found this specific form of prognostic conversation is delivered inconsistently due to multiple barriers, and the remaining research gaps are related to how and when these conversations should occur.

Our aim is to explore how prognostic conversations are currently taking place, what people think about prognostic conversations and how people experience them. We will conduct a qualitative synthesis using the JBI meta-aggregation approach, underpinned by pragmatic and Husserlian phenomenological philosophy.^18 19^ This protocol follows Preferred Reporting Items for Systematic review and Meta-Analysis Protocols (PRISMA-P) reporting guidelines^20^ and is prospectively registered with PROSPERO (CRD42024605240).

### Review question

What is the available evidence on the prognostic communication experiences, attitudes and practices of patients with heart failure and their healthcare professionals?

## Methods and analysis

The proposed review will be conducted in accordance with the JBI meta-aggregation approach for systematic reviews of qualitative evidence.^21^ Our review design will accommodate iterative changes, with any variance from this protocol reflected on PROSPERO and in the final manuscript. The start date of the review was 1 August 2024 and the expected completion date is 31 October 2025.

### Eligibility criteria

The following eligibility criteria were developed using the PICo mnemonic to define the population, phenomena of interest, and context for this review. In addition, the type of study has also been defined.

#### Inclusion Criteria

##### Population

The population of interest is adults, aged over 18 years, with heart failure of any cause, stage or classification, or health and social care professionals caring for this patient group. Data for patients and professional caregivers will be included from papers with other participants, such as family or other informal caregivers, if the patients and professional caregivers form at least half the participant sample (ie, 50% or above) and their data can be distinguished.

In studies where there is a mixed patient group in terms of diagnosis (eg, advanced life-limiting illness), they will be included if the heart failure patients are analysed and reported separately.

##### Phenomena of interest

This review will consider studies that explore prognostic communication, defined as a verbal interaction between two or more people (a patient and at least one healthcare professional) where they have discussed the expected journey of living with heart failure, which includes one or more of:

- Anticipated changes to symptoms.
- Anticipated changes to functional abilities and activities.
- The possibility of unpredictable events.
- Possible future treatments where there has been explicit use of prognosis in the interaction because either the patient’s prognosis will change with a treatment option, or the patient’s prognosis alters the treatment options available.
- Possible care at the end of life.
- Expression of wishes, hopes, worries and expectations about the future.
- Estimating life expectancy.
- Advance care planning.

Some studies may investigate interventions aiming to improve prognostic communication, such as question prompt lists, communication training or palliative care consultations. These will be included if the paper is clear and explicit that at least one of the aims of the intervention is to improve prognostic communication (as set out above).

For inclusion, studies need to explore one of:

- Experiences of prognostic communication—that is, how do people think, feel, react or talk about their own personal encounters of prognostic communication?
- Attitudes towards prognostic communication—that is, how do people think, feel and behave with regard to prognostic communication?
- Practices of prognostic communication—that is, how is prognostic communication undertaken in clinical practice?

##### Context

This study will consider prognostic communication occurring in any clinical or care setting. All geographic locations will be included to potentiate the learning from this review. Variations by geographical context will be included in the results and discussed in our analysis.

##### Types of studies

This review will consider studies that focus on qualitative data where the direct participant voice, fieldwork observations or other data are reported alongside the authors’ analytic interpretations.

Qualitative components of mixed-methods reviews will be included if the required data are able to be extracted from the published paper.

Other texts, such as reports or guidelines, will be included if they report empirical qualitative data on the prognostic communication experiences of included populations.

### Exclusion criteria

#### Population

Studies will be excluded if informal caregivers form the majority of the population (ie, above 50%), or their data cannot be separated from those of patient or professional caregivers.

#### Phenomena of interest

Studies focused on the deactivation of ICDs or assisted dying and/or euthanasia will be excluded.

#### Context

Studies published in languages other than English will be excluded based on resources available and the language proficiency of the authors. The number of articles excluded on this basis will be recorded, and where possible, any abstracts available in English will be assessed for potential relevance.

#### Types of studies

Studies that analyse text from clinical records or medical notes will be excluded.

Reviews, summaries and editorials will be excluded.

### Search strategy

The search strategy will use database and grey literature searches.

For database searches, search terms were developed iteratively with the support of an experienced university librarian using the concepts of heart failure, prognostic communication and qualitative research. Relevant text words and Medical Subject Headings (MeSH) index terms were identified from relevant articles identified using an initial limited search of MEDLINE (PubMed). These, alongside search strategies of other systematic reviews^4 22 23^ and a validated search filter to identify qualitative research,^24^ were used to develop a full search strategy for MEDLINE (Ovid) (see online supplemental material 1). The search strategy will then be adapted to search the following databases^25^: MEDLINE (Ovid), Excerpta Medica Database (EMBASE) (Ovid), PsycInfo (Ovid), Cumulative Index to Nursing and Allied Health Literature (CINAHL) Plus (EBSCOhost) and Web of Science Core. All databases will be searched from their inception date.

Overton, ProQuest Dissertations and Theses Global, and Google Scholar grey literature repositories^26^ will be searched using the terms heart failure, communication and prognosis. The first 200 results, sorted by relevance, will be considered.

Any reviews identified from database or grey literature searches that have an aim to explore an aspect of prognostic communication will have their reference list screened for additional relevant articles.

Prior to the final analysis, searches will be re-run, assisted by the use of database auto-alert systems.

### Study selection

Following the search, all identified citations will be collated, uploaded, managed and de-duplicated in Rayyan.^27^ Two independent reviewers will first screen titles and abstracts against the eligibility criteria, followed by full-text screening. Any disagreements that arise between the reviewers will be resolved through discussion or with a third reviewer. In the final systematic review, the search and study selection process, including reason for exclusion at full-text assessment, will be presented in a Preferred Reporting Items for Systematic Reviews and Meta-analyses (PRISMA) flow diagram.^28^

### Assessment of methodological quality

Two independent reviewers will critically appraise included studies for methodological quality using the standard JBI Critical Appraisal Checklist for Qualitative Research or Text and Opinion and its associated guidance.^18 21 29 30^ This will be supported by JBI System for the Unified Management, Assessment and Review of Information (JBI SUMARI; JBI, Adelaide, Australia).^31^

The JBI qualitative research critical appraisal tool is comprised of 10 questions that assess the following domains: congruity of philosophical perspective, methodology, research question, methods, representation of the data and interpretation of results; reflexivity of the researcher; representation of the participant’s voices and the conclusions drawn from them; and appropriate ethical approval. The JBI text and opinion critical appraisal tool consists of six questions. Each question can be scored as yes, no or unclear. Any disagreements that arise between the reviewers will be resolved through discussion or with a third reviewer.

All studies, regardless of the results of their methodological quality, will undergo data extraction and synthesis. The results of critical appraisal will be reported in narrative form and in a table. The implications of the critical appraisal will be considered in the analysis and discussion of the review.

There will be no assessments of meta-biases, such as publication bias across studies.

### Data extraction

Data will be extracted from studies included in the review using the standardised JBI data extraction tool using JBI SUMARI.^21^

One or two reviewers will perform data extraction with 10 studies as a pilot test. This process will highlight whether any changes are required to the data extraction tool. Data extraction of the remaining papers will be conducted by one reviewer, and if resources allow, a second independent reviewer will verify data extraction on a sample of 25%. Any questions or disagreements arising will be resolved through discussion with another independent reviewer.

The characteristics of each included study to be extracted will include: phenomenon of interest; methods for data collection and analysis; country; setting, context and culture; participant characteristics and sample size; and description of relevant main findings.

Study findings relevant to this review, and their illustrations, will be extracted verbatim and assigned a level of credibility. Findings are the authors’ interpretations reported in the manuscript, and illustrations are the primary data provided to evidence the finding. The level of credibility assigned to each paired finding and illustration can be assessed as unequivocal, credible or unsupported.

### Data synthesis

Findings and illustrations considered unequivocal and credible will be included in the synthesis. Findings that are unsupported by an illustration will be excluded. Qualitative research findings will be synthesised using JBI SUMARI, nVivo or Microsoft Word with the meta-aggregation approach.^18^

Papers will be graded as ‘high’, ‘medium’ or ‘low’ relevance in respect to answering the research question. Papers rated high and medium will be considered initially to form the preliminary synthesis. All papers will be read, and re-read, to harness and understand the meaning of the presented author interpretations. Extracted findings and their illustrations will be synthesised and categorised based on their similarity in meaning to produce a comprehensive set of synthesised findings. The low relevance papers will then be reviewed to incorporate and assess their alignment with the preliminary synthesised findings. Where textual pooling is not possible, the findings will be presented in narrative form.

### Assessing confidence in the findings

The confidence of the final synthesised findings will be graded according to the Confidence in the output of qualitative research synthesis (ConQual) approach and presented in a ‘Summary of Findings’.^32^

The ConQual approach was developed as a method to support assessment of confidence in findings of qualitative synthesis, similar to the Grading of Recommendations Assessment, Development, and Evaluation (GRADE) approach used for quantitative systematic reviews. The score for dependability is established from the five quality assessment questions on congruity of philosophical positioning, methodology and methods used. The score for credibility is assigned using the level of credibility of paired findings and illustrations. Together, the dependability and credibility scores determine the ConQual score of high, moderate, low and very low.

The Summary of Findings displays the synthesised finding alongside the type of research informing it, the dependability score, credibility score, overall ConQual score and any additional comments.

### Patient and public involvement statement

A group of 10 people with lived experience of heart failure (seven patients, two carers and one bereaved carer) are supporting a wider research project aiming to understand conversations in heart failure. For this review, they have helped shape its focus and research question. They will support dissemination of findings within their networks.

## Ethics and dissemination

Ethical approval is not required for the study since it is based on available published literature. Findings from the review will be disseminated through publication in a peer-reviewed journal. The MEDLINE (Ovid) search strategy is publicly available through PROSPERO, and all included papers are available in the public domain and will be referenced accurately in the final publication.

## Supplementary material

10.1136/bmjopen-2025-099088 online supplemental file 1
