# Supplementary material for: Prognostic communication in heart failure: protocol for a systematic qualitative synthesis of experiences, attitudes and practices
Source: BMJ Open. 2025 Nov 4;15(11):e099088. doi: 10.1136/bmjopen-2025-099088 (PMC12587945; doi:10.1136/bmjopen-2025-099088)
Supplement: online supplemental file 1 [file bmjopen-15-11-s001.docx]

### Supplemental Material 1: Search strategy

Database: MEDLINE (Ovid)

Search conducted on: 22 August 2024

| 1 | exp Heart Failure/ | 155507 |
| --- | --- | --- |
| 2 | ((heart or cardiac or myocardial or ventricular) adj3 (failure or incompetence or dysfunction or decompensation or insufficiency)).ti,ab,kf. | 301956 |
| 3 | exp Ventricular Dysfunction/ | 44602 |
| 4 | 1 or 2 or 3 | 352179 |
| 5 | (disease adj2 (course or trajector* or progression) adj3 (conversation* or understand* or communicat* or convey* or inform* or share* or sharing or talk* or interaction* or discuss* or exchang* or educat* or consult* or disclos* or awareness or counsel* or provid* or deliver*)).ti,ab,kf. | 3413 |
| 6 | ((palliative or terminal) adj3 (conversation* or understand* or communicat* or convey* or inform* or share* or sharing or talk* or interaction* or discuss* or exchang* or disclos* or awareness or counsel*)).ti,ab,kf. | 5999 |
| 7 | ((prognos* or uncertain* or trajector* or "serious illness" or ("end of life" or EOL) or ("goal* of care" or GOC)) adj3 (conversation* or understand* or communicat* or convey* or inform* or share* or sharing or talk* or interaction* or discuss* or exchang* or educat* or consult* or disclos* or awareness or counsel* or deliver*)).ti,ab,kf. | 42004 |
| 8 | ((estimat* or expec* or predict*) adj2 (survival or death or mortality or future or "life expectancy") adj3 (conversation* or understand* or communicat* or convey* or inform* or share* or sharing or talk* or interaction* or discuss* or exchang* or educat* or consult* or disclos* or awareness or counsel* or deliver*)).ti,ab,kf. | 1846 |
| 9 | exp Truth Disclosure/ | 15574 |
| 10 | (("do not attempt cardiopulmonary resuscitation" or "do not attempt resuscitation" or "do not resuscitate" or DNACPR or DNAR or DNR or withdraw*) adj3 (conversation* or understand* or communicat* or convey* or inform* or share* or sharing or talk* or interaction* or discuss* or exchang* or educat* or consult* or disclos* or awareness or counsel* or deliver*)).ti,ab,kf. | 1920 |
| 11 | (advance* care plan* or ((advance* or ("end of life" or EOL)) adj (decision* or directive* or statement* or plan or preference*)) or ACP).ti,ab,kf. or exp advance care planning/ or exp advance directive/ | 28649 |
| 12 | (shared decision making or SDM or ((shared or sharing or informed or collaborat* or cooperat* or support) adj (decision* or choice*))).ti,ab,kf. or exp decision making, shared/ | 39278 |
| 13 | (Disease Progression/ or Life expectancy/ or medical futility/ or treatment failure/ or prognosis/ or terminal care/ or survival analysis/ or palliative care/ or critical illness/ or Withholding Treatment/ or Treatment refusal/ or Attitude to Death/ or Attitude to Health/) and (Communication/ or Patient Education as Topic/ or professional-patient relations/ or disclosure/ or comprehension/ or Patient Participation/ or Attitude of Health Personnel/) | 35401 |
| 14 | 5 or 6 or 7 or 8 or 9 or 10 or 11 or 12 or 13 | 162438 |
| 15 | Qualitative Research/ | 91533 |
| 16 | Interview/ | 31227 |
| 17 | (theme$ or thematic).mp. | 194655 |
| 18 | qualitative.af. | 384372 |
| 19 | Nursing Methodology Research/ | 16427 |
| 20 | questionnaire$.mp. | 1021637 |
| 21 | ethnological research.mp. | 8 |
| 22 | ethnograph$.mp. | 15018 |
| 23 | ethnonursing.af. | 135 |
| 24 | phenomenol$.af. | 37427 |
| 25 | (grounded adj (theor$ or study or studies or research or analys?s)).af. | 16826 |
| 26 | (life stor$ or women* stor$).mp. | 1877 |
| 27 | (emic or etic or hermeneutic$ or heuristic$ or semiotic$).af. or (data adj1 saturat$).tw. or participant observ$.tw. | 33232 |
| 28 | (social construct$ or (postmodern$ or post-structural$) or (post structural$ or poststructural$) or post modern$ or post-modern$ or feminis$ or interpret$).mp. | 663241 |
| 29 | (action research or cooperative inquir$ or co operative inquir$ or co-operative inquir$).mp. | 6245 |
| 30 | (humanistic or existential or experiential or paradigm$).mp. | 210024 |
| 31 | (field adj (study or studies or research)).tw. | 20738 |
| 32 | human science.tw. | 270 |
| 33 | biographical method.tw. | 25 |
| 34 | theoretical sampl$.af. | 1012 |
| 35 | ((purpos$ adj4 sampl$) or (focus adj group$)).af. | 100722 |
| 36 | (account or accounts or unstructured or open-ended or open ended or text$ or narrative$).mp. | 888865 |
| 37 | (life world or life-world or conversation analys?s or personal experience$ or theoretical saturation).mp. | 18881 |
| 38 | ((lived or life) adj experience$).mp. | 22872 |
| 39 | cluster sampl$.mp. | 10244 |
| 40 | observational method$.af. | 1056 |
| 41 | content analysis.af. | 49087 |
| 42 | (constant adj (comparative or comparison)).af. | 6450 |
| 43 | ((discourse$ or discurs$) adj3 analys?s).tw. | 3329 |
| 44 | narrative analys?s.af. | 2167 |
| 45 | heidegger$.tw. | 813 |
| 46 | colaizzi$.tw. | 1420 |
| 47 | spiegelberg$.tw. | 88 |
| 48 | (van adj manen$).tw. | 594 |
| 49 | (van adj kaam$).tw. | 45 |
| 50 | (merleau adj ponty$).tw. | 276 |
| 51 | husserl$.tw. | 341 |
| 52 | foucault$.tw. | 996 |
| 53 | (corbin$ adj2 strauss$).tw. | 492 |
| 54 | glaser$.tw. | 1126 |
| 55 | 15 or 16 or 17 or 18 or 19 or 20 or 21 or 22 or 23 or 24 or 25 or 26 or 27 or 28 or 29 or 30 or 31 or 32 or 33 or 34 or 35 or 36 or 37 or 38 or 39 or 40 or 41 or 42 or 43 or 44 or 45 or 46 or 47 or 48 or 49 or 50 or 51 or 52 or 53 or 54 | 3137750 |
| 56 | 4 and 14 and 55 | 707 |
| 57 | limit 56 to english language | 681 |
