# Supplementary material for: Prognostic communication in heart failure: protocol for a systematic qualitative synthesis of experiences, attitudes and practices
Source: BMJ Open. 2025 Nov 4;15(11):e099088. doi: 10.1136/bmjopen-2025-099088 (PMC12587945; doi:10.1136/bmjopen-2025-099088)
Supplement: Supplementary file 2 [file bmjopen-2025-099088.reviewer_comments.pdf]

PEER REVIEW HISTORY

BMJ Open publishes all reviews undertaken for accepted manuscripts. Reviewers are asked to complete a checklist review form and are provided with free text boxes to elaborate on their assessment. These free text comments are reproduced below.

ARTICLE DETAILS

Title (Provisional)

Prognostic communication in heart failure: protocol for a systematic qualitative synthesis of experiences, attitudes, and practices.

Authors

Chu, Christina; Bloch, Steven; Yardley, Sarah

VERSION 1 - REVIEW

**Reviewer**	**1**
**Name**	**Atuhairwe, Irene**
**Affiliation**	**Stellenbosch University, Public Health**
**Date**	**02-Apr-2025**
**COI**	**None**

Include studies conducted in other languages as focusing on only English will miss out on key data.

The review question should be revised to say what is the available evidence on..... given that this study is looking at gathering the available data.

**Reviewer**	**2**
**Name**	**Godfrey, Sarah**
**Affiliation**	**UT Southwestern Medical Center**
**Date**	**16-Jun-2025**
**COI**	**None**

Understanding how we communicate prognosis to patients with heart failure is an essential research question, particularly outside the scope of end-of-life care. Conducting a systematic review is an interesting approach to understanding this question. I commend the authors on their ongoing work. Some suggestions for improvements:

1. The introduction is quite long and could likely be condensed for clarity and conciseness. The first 4 paragraphs, in particular, could be combined/shortened.

2. Why are all geographic locations included, rather than limiting the scope? There are likely significant variations based on country/region. Will this be discussed in the final manuscript?

3. It is unclear from the current manuscript -- will the healthcare professionals' qualitative experience with prognostic communication be examined? Or is this study focused soley on the patient? The caregivers' perspective is listed as one of the exclusion criteria -- does that mean that studies that include both caregiver and patient perspective will not be included?

VERSION 1 - AUTHOR RESPONSE

Reviewer 1 comments:

- We are unable to include studies published in languages other than English due to our available resources. The impact of this exclusion will be assessed and its limitations addressed in the study outputs. The review question has been amended as suggested by the reviewer.

Reviewer 2 comments:

- The introduction has been revised and condensed.
- All geographical locations have been included to enhance potential learning from this work. The geographical distribution of included studies, and any variations in their findings will be considered in our analysis and results.
- Clarification has been provided about the population eligibility criteria. Firstly, by distinguishing health and social care professional caregivers and informal caregivers, and secondly through greater detail on how studies with mixed participant groups are handled.
